# Time-to-Delivery after Maternal Transfer to a Tertiary Perinatal Centre

**DOI:** 10.1155/2014/325919

**Published:** 2014-03-18

**Authors:** Fiona H. Hutchinson, Mark W. Davies

**Affiliations:** ^1^Grantley Stable Neonatal Unit, Royal Brisbane and Women's Hospital, Brisbane, QLD 4029, Australia; ^2^Department of Neonatology, Mater Mothers' Hospital, Brisbane, QLD 4101, Australia; ^3^Department of Paediatrics & Child Health, The University of Queensland, Brisbane, QLD 4006, Australia

## Abstract

*Objectives*. To determine, in women transferred antenatally for acute admission with high risk pregnancies, the numbers who deliver, the average time from transfer to delivery, and whether the reason for transfer influences the time-to-delivery. * Methods*. A retrospective analysis of time-to-delivery was performed in a population of women transferred to the Royal Brisbane and Women's Hospital, QLD. Data were obtained from the hospital obstetric, neonatal, and admission databases. * Results*. A total of 941 women were transferred antenatally with high risk pregnancies where delivery was deemed potentially imminent. Of these 821 (87%) delivered at RBWH. The remaining 120 women (13%) were discharged prior to delivery and then delivered elsewhere. Of the 821 maternal transfers that delivered, the median time to delivery was 24.4 hrs. There were 43% who delivered within 24 hours of admission and 29% who either delivered after 7 days or delivered elsewhere. Most transfers for fetal abnormality delivered in the first 24 hours while most transfers for antepartum haemorrhage and preterm prelabour membrane rupture delivered beyond 24 hours. * Conclusion*. There are significant differences in time-to-delivery following transfer depending on the reason for transfer and many infants transferred *in utero* will not deliver imminently.

## 1. Introduction

Women with pregnancy complications, including fetal abnormalities, are frequently transferred to tertiary hospitals where specialist perinatal and neonatal care is available. Not all women transferred for the possibility of imminent birth will deliver immediately and not all high risk infants will need specialised neonatal management. In addition, a percentage of those potentially imminent deliveries will not deliver at all in that admission [1, 2].

It is widely accepted that antenatal transfer of these high risk pregnancies to a centre capable of providing optimal perinatal care is appropriate [3]. This is despite the uncertainty about the timing of delivery and despite the uncertainty of the need for specialist neonatal care if the infant does deliver during this admission. The need for transfer is supported by the research showing that neonatal outcomes of infants requiring neonatal intensive care management are better for those transferred* in utero* than those transferred as neonates [4–7].

Intensive care neonatal beds and staff are sometimes held at the receiving institution in anticipation of imminent high risk deliveries resulting in the blocking of neonatal beds [8]. At times antenatal transfers are delayed or refused because of the lack of availability of both a maternal bed and a neonatal bed within the same hospital [9]. This is becoming increasingly significant with the rising rates of preterm delivery and the resulting increase in demand for limited neonatal intensive care cots [10, 11]. There is little published data on the time-to-delivery following maternal transfer. Such information has significant potential to help with the management of staffing and beds in tertiary neonatal units which are frequently operating at or close to capacity.

The aim of this study was to review admissions of women, transferred antenatally for acute admission with high risk pregnancies, and determine the numbers who deliver, the average time from transfer to delivery (time-to-delivery), and whether the reason for transfer influences the time-to-delivery.

## 2. Methods

We report a retrospective analysis of time-to-delivery following maternal transfer for acute admission to a single tertiary perinatal centre over a 3-year time period (1/1/2009–31/12/2011).

The study population was all women who transferred antenatally for acute admission to the Royal Brisbane Women's Hospital, QLD. The RBWH is one of three tertiary hospitals in Queensland providing level 6 neonatal intensive care [12]. The neonatal unit has 30 intensive care cots and 39 special care cots.

Data were obtained by searching the hospital obstetric database for all* in utero* transfers where delivery occurred between 1/1/2009 and 31/12/2011. This data was cross-checked against data obtained from the hospital neonatal database of all infants born in this time period who were recorded as* in utero* transfers. Data of those women who were discharged prior to delivery could not be obtained from the obstetric or neonatal databases and this was obtained from the hospital admissions database, HBSCIS (Hospital Based Corporate Information System). Maternal information was collected on* in utero* transfer reason, place of origin, date and time of admission, gravidity, parity, and gestational age. Neonatal data was collected on date and time of birth and gestational age at delivery. The information available for those women who were discharged prior to delivery (obtained from HBSCIS) did not include transfer reason, place of origin, gravidity, parity, or gestational age.

### 2.1. Data Analysis

Summary statistics were calculated for the time from hospital admission to delivery (time-to-delivery) for the entire cohort and by groups based on the reason for transfer. Transfers were divided into nine groups according to the reason for transfer: antepartum haemorrhage (APH), fetal abnormality, intrauterine growth restriction (IUGR), maternal medical condition, PET, preterm prelabour membrane rupture (PPROM), preterm labour, unknown, and a miscellaneous group (see Table 1). The Kruskal-Wallis test was used to test for differences between median time-of-delivery and gestational age between the different groups. The posttest Dunn's multiple comparison test was applied to determine if there were any between group differences. Chi-squared test was used to test for differences in proportions. Analysis was performed using GraphPad Prism version 4.00 for Windows (GraphPad Software, San Diego, CA, USA).

## 3. Results

In this three-year time period a total of 941 women were identified as transferring antenatally, for acute admission, as a result of high risk pregnancies where delivery was considered to be potentially imminent. Searches of the neonatal and obstetric databases identified 821 of these and a further 120 were identified through the hospital admission database.

During the same time period, at the RBWH, there was a total number of 13 918 births from 13 581 mothers and a total of 4 750 admissions to the neonatal unit.

Of the entire cohort of 941 transferred women, 821 (87%) delivered at RBWH. The remaining 120 women (13%) were discharged prior to delivery and then delivered elsewhere, and for these admissions database information was limited to admission and discharge times and dates.

**Figure 1:**
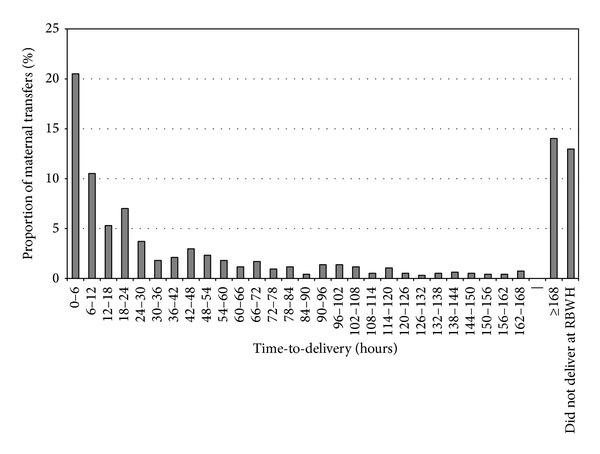
Time-to-delivery following* in utero* transfer for the entire cohort of 941 maternal transfers.

Of the entire cohort of 941 transferred women, the proportion who had either not delivered after 7 days or were discharged prior to delivery was 29% (see Figure 1). Delivery occurred within 6 hrs of admission in 21% of the transferred women and by 24 hrs in a further 23%.

Of those 821 women who delivered at RBWH the median time-to-delivery was 24.4 hrs (IQR 6.4–97.2 hrs). The shortest time-to-delivery was 0.02 hr and the longest time 2,060 hrs.

There were 30 transfers with more than one recorded reason for transfer. Multiple pregnancy was listed as one of two reasons for transfer for 20 of these. For these 20 the transfer was classified according to the other reason for transfer (e.g., if the reason for transfer was “twins/preterm labour” then the reason for transfer was classified as preterm labour). There were three transfers for APH/PPROM, all classified as PPROM; one APH/fetal abnormality classified as miscellaneous; one APH/PTL classified as PTL; one IUGR/fetal abnormality classified as fetal abnormality; one fetal abnormality/PTL classified as PTL; one PET/APH classified as PET; one PET/IUGR classified as PET; and one PPROM/PTL classified as PTL.

**Figure 2:**
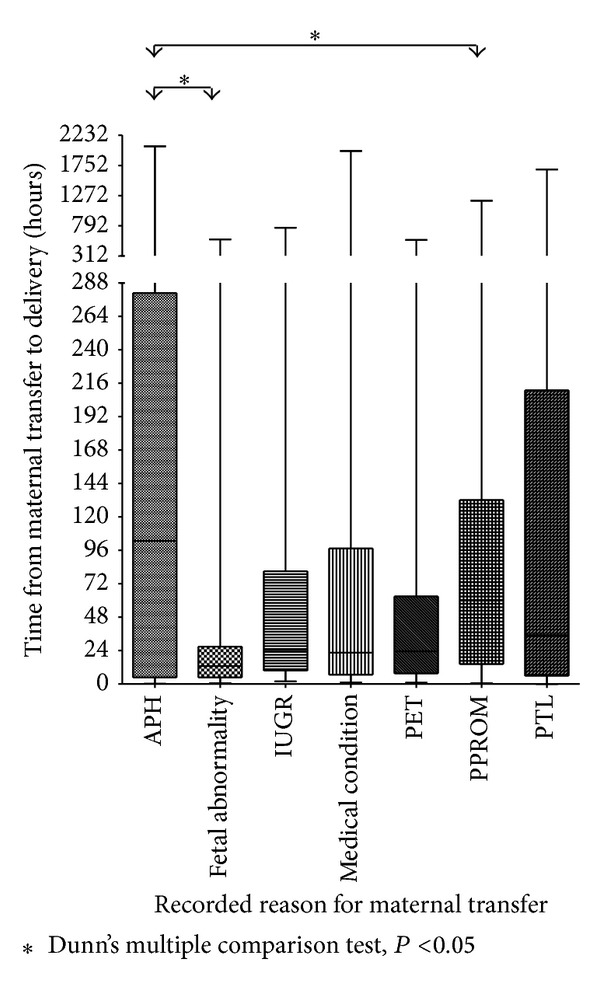
Primary reason for maternal transfer and associated time-to-delivery for the entire cohort of 941. The box identifies the median and 1st and 3rd quartiles, the whiskers identify the range.

Table 1 shows the grouping of the transferred women, the numbers in each group, and the median time-to-delivery for each group. The reason for transfer in the miscellaneous group included alloimmune thrombocytopenia, TTTS, fetal distress, hospital evacuation (due to a flood and a cyclone), rhesus isoimmunisation, oligohydramnios, and polyhydramnios. The differences between groups for median time-to-delivery were statistically significant (Kruskal-Wallis test, *P* value < 0.0001). Between group differences are shown in Figure 2.

Most transfers for fetal abnormality delivered in the first 24 hours while most transfers for APH and PPROM delivered beyond 24 hours (see Figure 2). The median time-to-delivery following transfer for fetal abnormality was 12.8 hours (IQR 4.76–26.85 hours), compared with a median of 60.0 hours for PPROM (IQR 14.38–132.10) and 102.8 hours for APH (IQR 4.88–280.7 hours).

Less than 35% of each group of women transferred with APH or PPROM delivered within the first 24 hours after admission and more than 55% of these women had not delivered by 72 hours. Greater than 65% of the women transferred because of fetal abnormality had delivered by 24 hours and less than 10% were still undelivered at 72 hours. In each of the groups transferred for IUGR, maternal medical condition, PET and PTL between 45 and 50% of the women had delivered by 24 hours and 25% or more were undelivered at 72 hours. The differences in proportions between the six groups were statistically significant (Chi-squared test *P* < 0.0001).

**Figure 3:**
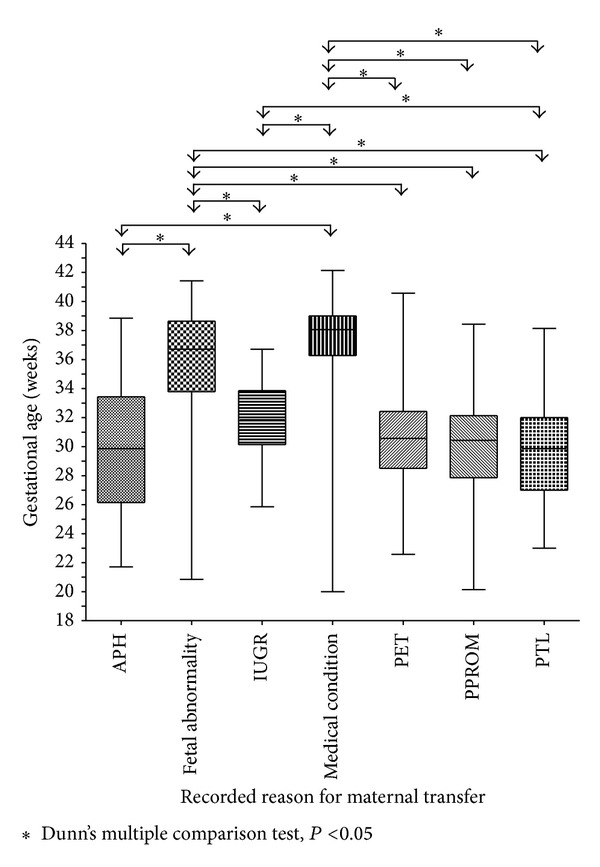
Primary reason for maternal transfer and associated gestational age at delivery. The box identifies the median and 1st and 3rd quartiles, the whiskers identify the range.

Mothers transferred because of fetal abnormalities or their own medical conditions had median gestational ages at delivery at or approaching term. The overwhelming majority of those transferred for all other reasons delivered preterm. See Table 1 and Figure 3.

## 4. Discussion

### 4.1. Summary of Findings and Interpretation of Results

Women with high risk pregnancies who are transferred acutely because of the likelihood of imminent delivery do not all deliver immediately. We found that the overall median time-to-delivery in our study population was 24.4 hrs, with 43% of women delivering within 24 hours and 29% either delivering after 7 days or being discharged and not delivering at the receiving tertiary hospital, RBWH. Most women transferred for fetal abnormality delivered within 24 hours and of those with APH and PET most delivered after 24 hours. It is possible that many with fetal abnormality were transferred with delivery already planned while those with APH and PET were more likely to be preterm and appropriate management may have been aimed at delaying delivery. Overall there was a large spread of times-to-delivery across almost all groups. This is not surprising given the fact that the overwhelming majority of women are transferred because of the threat of preterm delivery (or the need for it) as well as the desire to delay delivery as long as possible (if possible) to enhance maturity and give maternal steroids an opportunity to work.

Acute antenatal transfer allows for the provision of specialist neonatal care which is only available in a limited number of neonatal nurseries across Queensland. Previous studies have shown that neonatal outcomes of infants requiring neonatal intensive care are better for those transferred* in utero* than those transferred as neonates [4–6]. However, whilst this improvement in outcome following* in utero* transfer is well known for those babies born preterm and/or very low birth weight, those differences may well occur, at least in part, because of systematic differences between those babies transferred* in utero* and those where* in utero* transfer was not possible. Also, it is inevitable that by aiming to deliver these high risk pregnancies in a specialist neonatal unit there will continue to be some women who are transferred unnecessarily or earlier than necessary.

Gestational age at delivery was greater for the fetal abnormality and maternal medical condition groups. The overwhelming majority of those transferred for all other reasons delivered preterm and most of these were delivered before 32 weeks gestation; there were no clinically significant differences between these groups. APH, fetal growth restriction, preeclampsia, PPROM, and premature labour are all common causes of preterm delivery. In the absence of neonatal morbidity data the gestational age at delivery for each pregnancy is a surrogate marker of the need for NICU management (in all groups except fetal abnormality). In addition there were no clinically relevant differences in gravidity or parity between groups.

### 4.2. Strengths and Limitations

This study used data from a large group of women transferred antenatally to a single, large tertiary referral centre. All those women transferred for whatever reason were studied, unlike other cohorts that were restricted in some way (see next).

There are limitations when using database information. The reliability of the data from hospital and unit databases obviously depends on the voracity of the data entered into those databases. Verification of those data by looking at the clinical records would entail significantly more time and cost and was beyond the scope of this small observational study. For those women who did not deliver at RBWH there was a lack of clinical data available. For these women we were unable to obtain the reasons for transfer and thus unable to determine if there were a group or groups more likely to be discharged and not deliver in the receiving hospital.

### 4.3. Comparison with Other Studies

We are unaware of any other previous studies looking at times-to-delivery of all women transferred for acute admission,* for whatever reason*, antenatally within Australia, nor are we aware of any study that has determined whether the time-to-delivery differs by the reason for maternal transfer other than those specifically delivering preterm.

There are a number of published studies reporting the times-to-delivery following antenatal transfer and preterm delivery. Akl et al. performed a retrospective, observational study of aeromedical transfer of women at risk of preterm delivery in Western Australia and reported that 53% of the women transferred were discharged prior to delivery. The median time-to-delivery was 11 days (IQR, 2–32 days) for parous transfers and 3 days (IQR 1–18 days) for nulliparous transfers. They report that the clinical factors which were significantly associated with a shorter time-to-delivery were spontaneous preterm labour, ruptured membranes, cervical dilation, gestational age, nulliparity, contraction status, and corticosteroid administration [13].

A retrospective, observational study of antenatal transfers of rural women in NSW and ACT reports that 60% of women admitted after emergency transfer delivered in that admission [1]. A population-based cohort study which looked only at preterm hospital admissions in NSW (2001–2008) reported that of the women transferred to centres of higher care only 46% gave birth during that admission and the median time from maternal admission to birth was 2 days (IQR, 1–6 days). Transfer to another hospital occurred in 8.2% and almost half, 46%, of the women were discharged without giving birth [14]. This is almost twice the rate recorded by Fenton et al. [2] in a one-year prospective cohort study from northern England looking at all acute antenatal transfers between consultant obstetric units in a one-year period. This study reports that 24% of women did not deliver at the receiving hospital. The study did record the time-to-delivery in hours and found 19% delivered within 6 hours of admission and 31% within 48 hours [2]. There have been a number of other studies from the UK and America which have reported that between 15 and 40% of transferred women do not deliver within 7 days or leave hospital undelivered [3, 15, 16]. Behrenz et al. report on maternal-fetal transfers in Texas, USA, and describe 60% of women delivered during the transfer admission. They do not report on the time-to-delivery [16]. A Canadian study of emergency air transport of pregnant women found 63% delivered at the receiving hospital and 37% were discharged and delivered at their home hospital [17].

It is, however, difficult and possibly inappropriate to attempt to relate the UK, USA, and Canadian data and even other Australian data to Queensland due to the differences in our transfer and retrieval systems and the sometimes vast differences in geography. The study by Fenton et al. reported on antenatal transfers in the Northern Region of UK with a population of 3.2 million, 33 000 annual deliveries, and all long term neonatal care provided by four neonatal intensive care units [2]. This region covers an area of approximately 15 000 km^2^. In contrast the area covered for transfers and retrievals by the RBWH is greater than 250 000 km^2^ with 20 000–25 000 annual deliveries. The RBWH retrieval team is provided by the hospital NICU and staffed by experienced neonatal nurses and paediatric registrars trained for retrievals, familiar with the equipment used and under the phone guidance of experienced neonatologists. According to Fenton et al. postnatal transfer in the UK was frequently performed by staff who were using unfamiliar equipment and had limited guidance and experience in neonatal transfer [18]. Such differences in neonatal retrieval teams may impact on the decision to transfer antenatally. In addition some of the antenatal transfers described in the UK studies include transfers because of bed shortages [8]; this was not a reason for transfer in our study population. Each Australian state or territory has its own individual system for retrievals and transfer catering for its own particular needs. Other published Australian studies have looked only at threatened preterm deliveries and have all been conducted outside of QLD with different retrieval and transfer setups as well as geography.

### 4.4. Implications

The information from this study at the RBWH will assist in the planning of cots and staffing arrangements in the anticipation of possible deliveries of high risk infants following antenatal transfer. These data may also be useful in the planning for the provision and use of health care resources more generally.

Given the differences in the proportion of transfers that actually deliver in the tertiary unit and the differences by reason for transfer and the wide spread of timing of deliveries, it would seem appropriate for tertiary obstetric and neonatal units to do further regular audits of* in utero* transfers and time-to-delivery. This is especially important given the significant differences between countries (and regions within countries) in their* in utero* transfer policies and outcomes. These differences can also make comparisons between countries (and regions within countries) problematic.

## 5. Conclusion

This study has shown that there are significant differences in time-to-delivery following transfer depending on the reason for transfer and that significant numbers of infants transferred* in utero* will not deliver imminently. Previous Australian studies of women at risk of preterm delivery have reported similar findings.

## Figures and Tables

**Table 1:** Reasons for *in utero* transfer and associated median time-to-delivery for those that delivered at the RBWH.

Reason for transfer	Number of transfers (total *N* = 821) *N* (%)	Gravidity Median (IQR)	Parity Median (IQR)	Time-to-delivery (hours) Median (IQR)	GA at delivery (weeks) Median (IQR)
Antepartum haemorrhage	75 (9.1%)	4 (2–6)	1 (0–3)	102.8 (0.2–280.7)	29.86 (26.14–33.43)
Fetal abnormality	89 (10.8%)	2 (1–4)	1 (0–2)	12.8 (0.6–26.8)	36.71 (33.79–38.64)
Intrauterine growth restriction	62 (7.5%)	3 (1–5)	1 (0–2)	24.3 (1.9–81.1)	31.86 (30.14–33.86)
Maternal medical condition	95 (11.5%)	3 (1–4)	1 (0–2)	22.6 (6.8–97.3)	38.07 (36.29–39.00)
Preterm prelabour membrane rupture	175 (21.3%)	2 (1–4)	1 (0–2)	60.0 (14.4–132.1)	30.43 (27.86–32.14)
PET	97 (11.8%)	2 (1–3)	0 (0-1)	23.5 (7.7–62.8)	30.57 (28.50–32.43)
Preterm labour	141 (17.1%)	2 (1–4)	1 (0-1)	35.1 (6.1–210.8)	29.86 (27.00–32.00)
Miscellaneous group	64 (7.8%)	3 (2–4)	1 (0–2)	6.8 (3.6–21.5)	34.43 (31.14–37.5)
Unknown	26 (3.1%)	2 (1–4.5)	1 (0–2)	9.0 (3.9–20.0)	30.67 (26.94–33.37)
